# Impact of preoperative ultrasonography findings on the patency rate of vascular access in Japanese hemodialysis patients

**DOI:** 10.1186/s40064-016-2082-z

**Published:** 2016-04-14

**Authors:** Junichiro Nakata, Hiroaki Io, Tomonari Watanabe, Yu Sasaki, Yuko Makita, Tatsuya Aoki, Hiroyuki Yanagawa, Reo Kanda, Yasuhiko Tomino

**Affiliations:** Division of Nephrology, Department of Internal Medicine, Juntendo University Faculty of Medicine, 2-1-1 Hongo, Bunkyo-ku, Tokyo, 113-8421 Japan; Medical Corporation SHOWAKAI, Tokyo, Japan

**Keywords:** Vascular access (VA), Arteriovenous fistula (AVF), Patency rate, Ultrasonography, Hemodialysis, Radial artery

## Abstract

**Background:**

Although ultrasonography before a vascular access (VA) operation has become popular in recent years, benchmarks for the diameter or blood flow of arteries or veins are not defined in Japan. The objective of the present study is to analyze the relationship between preoperative US findings and the patency rate of VA in Japanese hemodialysis patients.

**Methods:**

139 patients with end stage kidney disease were enrolled in this study. They had been given primary radiocephalic arteriovenous fistula (AVF) from February 2009 to December 2010 at the Juntendo University Hospital and were followed up over 2 years. We defined the interval from the time of AVF creation until first access thrombosis or any intervention to maintain or restore blood flow as patency time (primary patency). We examined the correlation between the 2-year primary patency rate of VA and the diameter of the radial artery (RA), brachial artery (BA), or cephalic vein at an anastomosis presumptive region by US, the blood flow of RA or BA, as measured by US, age, gender, and primary kidney diseases.

**Results:**

The average patency term was 448.6 ± 271.3 days, with the 1-year and 2-year patency rate as 64.0 and 51.2 %, respectively. The patency rate was significantly lower in elderly patients over the age of 75 and in patients with diabetes mellitus. US findings of 2.0 mm or less in the RA diameter also resulted in a noticeably low patency rate. A multivariate analysis indicated that those factors were risk factors for early VA failure.

**Conclusions:**

Preoperative US findings of the diameter of RA may involve the patency rate of VA, making it appears that an RA of 2.0 mm or more in diameter at an anastomosis region may be more effective for the improvement in the patency rate of VA.

## Background

Recently, patients on long-term hemodialysis (HD) therapy have been increasing in Japan. Maintenance of adequate vascular access (VA) for HD is a major problem from the standpoint of quality of life in end stage kidney disease (ESKD) patients. A native arteriovenous fistula (AVF) is the most preferable form of VA (National Kidney Foundation Kidney Disease Outcomes Quality Initiative 2006; Tordoir et al. 2007). VA failure remains the most common cause of morbidity and hospitalization in HD patients. Adequate delivery of prescribed HD relies on an optimally functioning VA. Therefore, it is very important to create a long-term-functioning VA.

Ultrasonography (US) is a mobile, simple and noninvasive technique that provides both anatomic (blood vessel lumen and wall) and physiologic (flow measurement) data. Although physical examinations have traditionally been used to identify a suitable artery and vein for AVF formation (Lok et al. 2005) and a very recent randomized controlled trial (RCT), comparing a selective and a routine policy of US before AVF surgery, found no significant differences in primary patency and complication rates (Smith et al. 2014), US before VA operation has become popular in recent years and is recommended in some guidelines (National Kidney Foundation Kidney Disease Outcomes Quality Initiative 2006; American College of Cardiology Foundation Appropriate Use Criteria Task Force et al. 2013). It is considered that the gap between the value of preoperative evaluation and the missing maturation mainly depends on the experience of the VA surgeon (Konner et al. 2013) and preoperative US may help the VA surgeon to plan the most appropriate AVF configuration, potentially reducing the incidence of VA dysfunctions (Caroli et al. 2013; Lomonte and Basile 2015). It was reported that preoperative US improves AVF outcomes (Silva et al. 1998; Malovrh 1998; Allon et al. 2001; Ferring et al. 2010) and results in a marked increase in the placement of AVFs, as well as an improvement in the adequacy of forearm fistulas for HD (Allon et al. 2001; Mendes et al. 2002). In those studies, patients with a minimum cephalic vein (CV) diameter of 2–2.5 mm and a minimum arterial diameter of 2 mm showed a significantly higher incidence rate of functional fistula maturation (Allon et al. 2001; Mendes et al. 2002). However, the relationship between US findings, including the diameter of vessels or blood flow of artery and patency rate of AVF, has not been reported in Japanese HD patients.

The objective of the present study is to analyze the relationship between patency rate of VA and preoperative US findings including the diameter and the blood flow of both arteries and veins, and to discuss the clinical benefit of US before VA operation in Japanese HD patients.

## Results and discussion

### Results

#### Patient characteristics

The baseline characteristics of all Japanese patients enrolled in this study are shown in Table 1. The average of age at VA operation was 65.3 ± 12.3 years old and 65.5 % of all patients were male (91/139). The etiology of 42 % of all patients was diabetes mellitus (DM) (58/139). The average body weight as measured on the morning of the day of operation was 58.40 ± 12.47 kg and body height was 160.13 ± 8.71 cm. The average blood pressure as measured in the supine position just before operation was 163.83 ± 24.92/83.70 ± 14.13 mmHg. The average duration between VA operation and first puncture was 46.56 ± 76.17 days.

**Table 1:** Patient characteristics

Age at VA operation (years old)	65.3 ± 12.3 (31–90)
Gender (male %, male:female)	65.5 (91:48)
Etiology of ESKD (DM %, DM:non-DM)	41.7 (58:81)
Body height (cm)	160.13 ± 8.71 (142.6–186.1)
Body weight (kg)	58.40 ± 12.47 (33.3–105.9)
Body mass index (kg/m^2^)	22.64 ± 3.77 (15.30–36.98)
Systolic blood pressure (just before operation) (mmHg)	163.83 ± 24.94 (112–222)
Diastolic blood pressure (just before operation) (mmHg)	83.70 ± 14.13 (60–140)
Duration between operation to puncture (days)	46.56 ± 76.12 (6–652)
Patency time (days)	448.6 ± 271.3 (20–730)

Data are presented as mean ± standard deviation

*VA* vascular access, *ESKD* end stage kidney disease, *DM* diabetes mellitus

#### US findings previous to the VA operation

The average diameter of CV and radial artery (RA) at an anastomosis presumptive region was 2.55 ± 0.69 and 2.57 ± 0.50 mm, respectively, and the average RA blood flow at an anastomosis presumptive region was 25.94 ± 18.74 ml/min. The average diameter and blood flow of brachial artery (BA) at the elbow was 4.82 ± 0.75 mm and 87.32 ± 52.52 ml/min, respectively (Table 2).

**Table 2:** Ultrasonography findings prior to the operation

Diameter of CV (mm)	2.55 ± 0.69 (0.96–4.85)
Diameter of RA (mm)	2.57 ± 0.50 (1.40–4.21)
Blood flow of RA (ml/min)	25.94 ± 18.74 (2.45–147.8)
Diameter of BA (mm)	4.82 ± 0.75 (3.19–7.40)
Blood flow of BA (ml/min)	87.32 ± 52.52 (4.86–320.25)

Data are presented as mean ± standard deviation

*CV* cephalic vein, *RA* radial artery, *BA* brachial artery

#### Patency rate

**Fig. 1:**
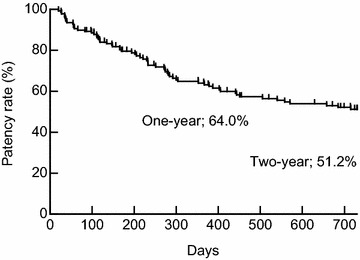
Cumulative primary patency for AVF. The average patency time was 448.6 ± 271.3 days. The 1-year and 2-year patency rate were 64.0 and 51.2 %, respectively (n = 139)

The average patency time was 448.6 ± 271.3 days. The 1-year and 2-year patency rate was 64.0 and 51.2 %, respectively (Fig. 1).

**Fig. 2:**
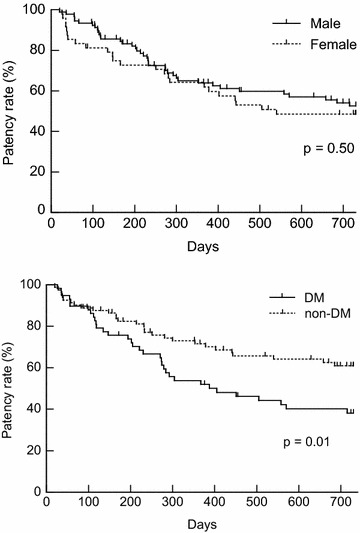
The *upper panel* shows the result of a comparison between male and female patency rates. No significant difference was shown in the patency rate between both genders (male, *solid line*, n = 91; female, *dotted line*, n = 48) (p = 0.50). The result of comparison between DM and non-DM patients in the patency rate is indicated in the *lower panel*. The patency rate in patients with DM (*solid line*, n = 58) was significantly lower than patients without DM (*dotted line*, n = 81) (p = 0.01)

**Fig. 3:**
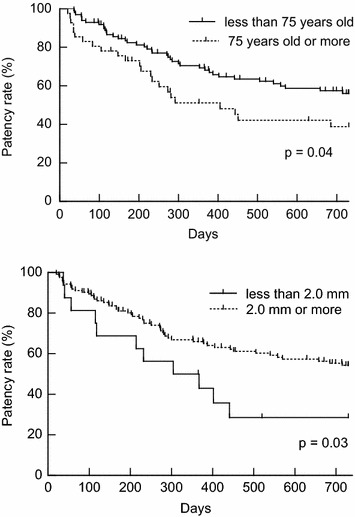
The *upper panel* shows the relationship between age and patency rate. The patency rate was also significantly lower in 75 years or older patients (*dotted line*, n = 41) than less than 75 years old patients (*solid line*, n = 98) (p = 0.04). Relationship between diameter of RA and patency rate is indicated in the *lower panel*. US findings of 2.0 mm or less in RA diameter (*solid line*, n = 11) are also resulted in a significantly lower patency rate than that of more than 2.0 mm (*dotted line*, n = 128) (p = 0.03)

No significant difference was shown in the patency rate between male and female patients (upper panel of Fig. 2, p = 0.50). By contrast, the patency rate in patients with DM (n = 58) was significantly lower than that in patients without DM (n = 81) (lower panel of Fig. 2, p = 0.01). Furthermore, the patency rate was also significantly lower in 75 years or older patients (n = 41) (upper panel of Fig. 3, p = 0.04). US findings of 2.0 mm or less in RA diameter also resulted in significant low patency rate (n = 11) (lower panel of Fig. 3, p = 0.03).

#### Contribution factors to the patency rate of VA

In the univariate analysis, it was indicated that age (75 or more years old, hazard ratio (HR) 1.72; 95 % confidence intervals (95 % CI) 1.02–2.85), etiology of ESKD (with DM, HR 1.86; 95 % CI 1.13–3.07), RA diameter (less than 2.0 mm, HR 2.76; 95 % CI 1.26–5.37), RA blood flow (less than 20 ml/min, HR 2.04; 95 % CI 1.24–3.40) and BA blood flow (per 1.0 ml/min increase, HR 0.99; 95 % CI 0.99–1.00) were the risk factors to early AVF failure (Table 3). By contrast, there was no correlation between poor prognosis of VA and gender (female, HR 1.19; 95 % CI 0.71–1.97), physical constitution (body height per 1 cm increase, HR 0.98; 95 % CI 0.96–1.01; body weight per 1 kg increase, HR 1.00; 95 % CI 0.98–1.02, body mass index per 1 kg/m^2^ increase, HR 1.03; 95 % CI 0.97–1.09), blood pressure (systolic per 1 mmHg increase, HR 1.00; 95 % CI 0.99–1.01, diastolic per 1 mmHg increase, HR 0.99; 95 % CI 0.97–1.00), duration from operation to puncture (per 1 day increase, HR 1.00; 95 % CI 0.99–1.00) and CV diameter (per 1.0 mm increase, HR 0.86; 95 % CI 0.59–1.22).

**Table 3:** Contribution factors to the patency rate

	Univariate analysis		Multivariate analysis	
	Hazard ratio (95 % CI)	p value	Hazard ratio (95 % CI)	p value
Age (per 1 year)	1.02 (1.00–1.04)	0.0607		
75 or more years old (vs. <75 years old)	1.72 (1.02–2.85)	0.0440*	2.14 (1.21–3.72)	0.0093*
Female (vs. male)	1.19 (0.71–1.97)	0.502		
DM (vs. without DM)	1.86 (1.13–3.07)	0.0145*	1.82 (1.11–3.02)	0.0371*
Body height (per 1 cm increase)	0.98 (0.96–1.01)	0.232		
Body weight (per 1 kg increase)	1.00 (0.98–1.02)	0.930		
Body mass index (per 1 kg/m^2^ increase)	1.03 (0.97–1.09)	0.345		
Systolic blood pressure (per 1 mmHg increase)	1.00 (0.99–1.01)	0.939		
Diastolic blood pressure (per 1 mmHg increase)	0.99 (0.97–1.00)	0.132		
Duration between operation to puncture (per 1 day)	1.00 (0.99–1.00)	0.487		
CV diameter (per 1.0 mm increase)	0.86 (0.59–1.22)	0.402		
RA diameter (per 1.0 mm increase)	0.60 (0.34–1.00)	0.0517		
Less than 2.0 mm (vs. 2.0 mm or more)	2.76 (1.26–5.37)	0.0133*	2.83 (1.18–6.06)	0.0215*
RA blood flow (per 1.0 ml/min increase)	0.98 (0.96–0.99)	0.0060*	0.28 (0.01–7.23)	0.485
Less than 20 ml/min (vs. 20 ml/min or more)	2.04 (1.24–3.40)	0.0048*	1.21 (0.60–2.62)	0.626
BA diameter (per 1.0 mm increase)	0.78 (0.55–1.10)	0.162		
BA blood flow (per 1.0 ml/min increase)	0.99 (0.99–1.00)	0.0041*	0.23 (0.02–2.29)	0.221

*CI* confidence interval, *DM* diabetes mellitus, *CV* cephalic vein, *RA* radial artery, *BA* brachial artery

* p value <0.05

The multivariate analysis revealed that those age of 75 or more years (HR 2.14; 95 % CI 1.21–3.72), with DM (HR 1.82; 95 % CI 1.11–3.02), or with less than 2.0 mm of RA diameter (HR 2.83; 95 % CI 1.18–6.06) were at major risk factor to early AVF failure, whereas RA blood flow (HR 1.21; 95 % CI 0.60–2.62) and BA blood flow (HR 0.23; 95 % CI 0.02–2.29) were not.

### Discussion

Many papers have reported the patency rate of VA, and in meta-analysis, the 1-year and 2-year primary patency rate of AVF created at the forearm, excluding primary AVF failure, were 62 and 51 %, respectively (Al-Jaishi et al. 2014). In this study, the 1-year and 2-year patency rate was 64.0 and 51.2 %, respectively; therefore our technical levels of VA operation fall in line with the average and are not poor.

The patency rate of this study was significantly lower in elderly patients and patients with DM. We have reported that AVF blood flow gradually increases after operation (Sato et al. 2012). In both elderly and DM patients, the progression of arteriosclerosis might have an influence on the postoperative increment of blood flow and it might affect the patency rate. Because the main volume of newly started HD patients are in their 70 s in Japan, we have divided patients into before and after the age of 75 and analyzed them. In the univariate analysis of this study, it was indicated that at the age of 75 or more, diabetic nephropathy, RA diameter of less than 2.0 mm, RA blood flow of less than 20 ml/min, and BA blood flow were risk factors for early AVF failure. In particular, depending on the US findings, the RA diameter, RA blood flow, and BA blood flow might influence the patency rate. However, the multivariate analysis has indicated that just an RA diameter of less than 2.0 mm was a risk factor for early AVF failure, dependent on US findings. This study has indicated that the blood flow of RA or BA might not affect the patency rate. Although it was reported that an RA blood flow of 20–40 ml/min might be associated with better outcomes of AVF (Sato et al. 2012; Yerdel et al. 1997), a benchmark of blood flow of BA is not defined. In the univariate analysis, RA and BA blood flow were significantly correlated to the patency rate. The RA diameter was a stronger contribution factor to the patency rate in multivariate analysis. A CV diameter of 1.6–2.5 mm with a tourniquet was recommended as an anastomosis region in some reports (Silva et al. 1998; Mendes et al. 2002; Malovrh 2002; Wong et al. 1996). However, in this study, no relationship between CV diameter and the patency rate of AVF was observed. Since the average diameter of CV in this study was more than 2.5 mm, the largeness of the CV diameter might affect this result. Moreover, one report suggested that venous distensibility is a predictor of AVF success, whereas luminal diameters are not (van der Linden et al. 2006). Other venous factors except for diameter might affect the patency rate. The present study suggests that measurements of RA that are 2.0 mm or more in diameter at an anastomosis region measured by US are more effective for the improvement in the patency rate of VA, in common with many papers that have reported that more than a 2.0 mm RA diameter was a successful factor for function or prognosis of VA (Allon et al. 2001; Mendes et al. 2002). It has been reported that patency rate of VA was significantly lower in female (Kazemzadeh et al. 2012). Interestingly, no significant difference regarding gender was observed in this study, while the RA diameter in females was significantly smaller than that in male patients (p = 0.0081). By contrast, age (p = 0.47) and CV diameter (p = 0.86) were not different between genders. Because of the smaller population of DM in female patients (p = 0.07), gender might have no discernible impact on the patency rate, despite a smaller RA diameter.

US is a mobile, simple and noninvasive technique that provides both anatomic and physiologic data. Many European and American papers have suggested that preoperative arterial and venous mapping by the B-mode method using US is useful for reducing the failure rate of forearm fistulas. Moreover, the B-mode and color Doppler methods can successfully detect stenosis, thrombi, and aneurysms. The pulse Doppler method is also useful for the observation of blood stream patterns and the measurement of blood flow and blood volume in order to analyze VA (Allon et al. 2001; Noto and Noto 2008). Similar to these reports, our results suggested that a preoperative US examination may allow for a better prognosis of VA in Japanese HD patients. Although the 2011 update Japanese Society for Dialysis Therapy Guidelines of Vascular Access Construction and Repair for Chronic Hemodialysis recommends a US examination before VA operation (Kukita et al. 2011), the relationship between patency rate of VA and US in Japanese HD patients has not been reported. In contrast, many papers from Europe or USA had reported this relationship. Therefore, the current findings provide important clues for establishing the clinical utility of US for VA in Japanese patients. Further studies to analyze factors involving the patency rate, such as vessel quality including arteriosclerosis or arterial calcification, surgical expertise, or dialysis prescription, are required. Moreover, further long-term observation is also required.

Nephrologists should make critical decisions regarding the choice of renal replacement therapy or take the initiative in performing VA-related procedures themselves. It has been reported that minimized delays, decreased hospitalizations, and decreased use of temporary catheters, and thereby improved medical care, decreased costs, and increased patient convenience, has been made by nephrologists in the performance of HD access-related procedures (Asif et al. 2007). The American Society of Diagnostic and Interventional Nephrology (ASDIN) has established guidelines for training, certification, and accreditation of HD vascular access and endovascular procedures (American Society of Diagnostic and Interventional Nephrology 2003). Recommendations for the training of European pediatric nephrologists by the European Society for Pediatric Nephrology define pediatric nephrologists as those who can acquire the application of HD, peritoneal dialysis (PD), and related techniques (European Society for Paediatric Nephrology 2001). The trainees should acquire skills for the application of HD and related techniques, together with vascular access for acute and chronic problems (Phadke and Bagga 2005). Nephrology practices are now moving toward total dialysis care. In our opinion, which is similar to this trend, nephrologists who follow patients with ESKD must act as interventional nephrologists. Therefore, skills in VA-related procedures, the same as PD-related procedures (Io et al. 2014), and in the same way as other interventions related to ESKD, must be part of the training of nephrologists.

## Conclusions

In conclusion, it appears that the patency rate was significantly lower in elderly patients and patients with DM. Moreover, preoperative US findings of the diameter of RA may involve the patency rate of VA, making it appears that an RA of 2.0 mm or more in diameter at an anastomosis region may be more effective for the improvement in the patency rate of VA in Japanese HD patients.

## Methods

We designed a cohort study to provide clinical evidence of the efficacy of a US examination before VA operation. All procedures performed in studies involving human participants were in accordance with the ethical standards of the institutional and/or national research committee and with the 1964 Helsinki declaration and its later amendments or comparable ethical standards and the protocol was approved by the Ethics Committee of Juntendo University Hospital, Tokyo, Japan. Informed consent was obtained from all individual participants included in the study.

### Patients

The inclusion criteria for our cohort were patients who had been given a primary radiocephalic arteriovenous fistula (RCAVF) in the forearm from February 2009 to December 2010 at the Juntendo University Hospital, Tokyo, Japan and had been followed up for 24 months (n = 146). Moreover, all of RCAVF were created by side-to-side anastomosis with the closure of the distal end of the vein (considered functionally equivalent to the side (artery) to end (vein) anastomosis). An exclusion criterion was patients with primary AVF failure, including immediate failure or early thrombosis within 7 days (n = 7) (Allon and Robbin 2002; Miller et al. 2003; Mihmanli et al. 2001).

In total, 139 Japanese patients with ESKD were enrolled in this study. Patient characteristics are shown in Table 1.

### Preoperative assessment

All patients enrolled in this study were evaluated with a physical and US by nephrologists who performed the AVF operation. In the physical examination, the pulses at the elbows and wrists, and the superficial veins in the forearm and upper arm with tourniquet were assessed. Vessels were considered suitable if the artery had a good pulsation and the vein was patent and of good caliber. US was performed based on a standardized protocol using a LOGIQ e or LOGIQ P5 (GE Healthcare Japan, Tokyo, Japan) and a 5–13 MHz linear transducer (12L-RS, GE Healthcare, Tokyo, Japan) with patients in a recumbent position.

After the application of a tourniquet, the superficial veins were followed in cross section with B-mode from the mid-upper arm to wrist with intermittent vein compression. The CV was scanned from mid-upper arm to wrist. The basilic vein was scanned from the elbow level to its drainage into the deep brachial veins. Internal diameters and vein depth were measured at an anastomosis presumptive region.

The arterial scan followed the vasculature from the BA at the elbow to the RA at the wrist in B-mode. Internal diameters and depth of the RA were measured at an anastomosis presumptive region and of the BA at the elbow. Longitudinal color flow images were obtained from the BA at the elbow to the RA at the wrist. Waveforms were recorded from a small sampling volume placed in the central flow stream at the attempted angles of 60° relative to vessel walls of the artery. Velocity sampling was done at RA in an anastomosis presumptive region and BA in the elbow by pulse Doppler mode.

### Surgical technique

The operation was performed under local anesthesia with 1 % lidocaine containing 0.001 % epinephrine. A light angle incision was made. Both the CV and the RA were isolated as distally as possible on the forearm. The CV was dilated by injecting heparinized saline. The end of the CV was ligated and the sidewall of CV was anastomosed to the sidewall of the RA using 6-0 polypropylene in a smooth loop configuration with a diameter of 6 mm. The suturing was initiated with posterior sutures, followed by anterior sutures. The operators did not use the microscope.

### Follow up

We contacted by mail the satellite dialysis clinic where patients who had undergone an operation went to receive HD therapy from December 2012 to January 2013. We obtained information about AVF failure, including the date of first access thrombosis or any intervention to maintain or restore blood flow, mortality or kidney transplant.

We defined the interval from the time of AVF creation until first access thrombosis or any intervention to maintain or restore blood flow as patency time (primary patency) (Lee et al. 2011; Sidawy et al. 2002).

### Statistical analysis

The patency rate was estimated using the Kaplan–Meier technique. The difference in the patency rate between the two groups was examined using a log-rank test. A univariate analysis of categorical variables was made with a Chi squared test and a multivariate analysis of AVF survival was examined by the Cox proportional-hazards model. Data were expressed as mean ± standard deviation (M ± SD). p < 0.05 was considered significant. All statistical analyses were performed using the Windows version of JMP 7.0.2 software (SAS Institute Inc., Cary, NC, USA).

## Abbreviations

US: ultrasonography
VA: vascular access
AVF: arteriovenous fistula
RA: radial artery
BA: brachial artery
CV: cephalic vein
HD: hemodialysis
ESKD: end stage kidney disease
RCT: randomized controlled trial
DM: diabetes mellitus
HR: hazard ratio
CI: confidence intervals
ASDIN: The American Society of Diagnostic and Interventional Nephrology
PD: peritoneal dialysis
RCAVF: radiocephalic arteriovenous fistula
